# Two Sides of a Coin: Molecular, metabolic, and Phenotypic Convergence in Pediatric Undernutrition and Obesity

**DOI:** 10.1007/s13679-026-00689-5

**Published:** 2026-03-02

**Authors:** Mercy Eloho Sosanya, Jennifer L. Temple

**Affiliations:** Department of Exercise and Nutrition Sciences, School of Public Health and Health Professions, University at Buffalo, Buffalo, NY USA

**Keywords:** Severe acute malnutrition, Pediatric obesity, Epigenetics, Immune disorders, Gut microbiota, Endocrine pathways

## Abstract

**Purpose of Review:**

This narrative review juxtaposes the metabolic and molecular consequences of pediatric under- and overnutrition, highlighting the similarities and differences between these two nutritional states occurring simultaneously in different parts of the world.

**Recent Findings:**

Numerous biological changes in pediatric acute undernutrition and obesity have been linked to elevated risks of chronic metabolic disorders. We summarize recent evidence on pathophysiological pathways and outcomes common to both conditions. Despite etiological divergence, early-life nutritional imbalances converge on shared mechanisms and consequences with intergenerational implications.

**Summary:**

Both acute undernutrition and obesity in childhood have intersecting long-term outcomes including insulin resistance, type 2 diabetes, cardiovascular diseases, hepatic steatosis, cancers, and others, mediated through endocrine, immunological, epigenetic, and gut microbiome pathways, albeit via diverse specific mechanisms. Robust, longitudinal studies in varied geopolitical settings are needed to further elucidate the complex mechanisms, long-term phenotypic consequences, and therapeutic effects in these twin conditions.

## Introduction

Malnutrition, which refers to an imbalance between nutrient needs and intake or utilization, encompasses undernutrition (wasting, stunting, underweight or micronutrient deficiencies), and overweight or obesity, with their resulting adverse sequelae [1]. Undernutrition and overnutrition have interconnected, underlying mechanisms, despite overt differences [1, 2]. Almost 45% of mortality in children < 5 years in the Global South is imputable to undernutrition, translating to 2.9 million child deaths annually [3, 4]. In 2024, acute malnutrition, a state of nutritional insufficiency from protein or energy deficits, affected 42.8 million children < 5 years [5]. Of these, 12 million children experienced severe acute malnutrition (SAM), which has an 8–35% case fatality rate even when treated [5, 6].

SAM is characterized as a weight-for-height *z*-score (WHZ) < − 3SD or a mid-upper arm circumference (MUAC) lower than 115 mm, with/without bilateral edema. In addition to diets deficient in macro- and micronutrients, acute childhood illnesses, and suboptimal infant and young child feeding practices also contribute to the etiology of SAM [7, 8]. This disorder clinically manifests as edematous SAM (ESAM or kwashiorkor, with bilateral edema and 60–80% of expected body weight), non-edematous SAM (NESAM or marasmus, with < 60% of expected body weight) or marasmic-kwashiorkor [9]. Numerous metabolic changes in SAM result in altered phenotypes with immediate and long-term adverse health outcomes (described in detail below) [10]. 

On the other hand, obesity denotes excess energy consumption above requirements or expenditure, characterized by inordinately high body fat accumulation that elevates disease risk [11, 13] Globally, over 35 million children < 5 years of age are affected by overweight and obesity, defined by weight-for-height Z-scores > 2 SD or > 3 SD, respectively, of the WHO growth reference (2006) [14, 15] Childhood obesity has been linked to cerebrovascular diseases [16, 17] type 2 diabetes [18–20], a 33% increase in cancer incidence in adulthood [21], and markedly elevated prevalence ratios for cardiometabolic complications, ranging from 21.2 to 58.0 [22] for cardiovascular risk factors and 26.1 [22] for non-alcoholic fatty liver disease.

The escalating burden of pediatric obesity is broadly attributable to genetics, prenatal factors (maternal diets, weight, chronic disease, smoking and other toxins), and postnatal influences (unhealthy infant and young child feeding practices, excessive energy intake, altered sleep cycles, increased sedentary/screen time and snacking patterns) [13, 23, 24]. Also, changes in the structural environment which limit access to nourishing food and recreational spaces, and family and parenting demographics and dynamics contribute to this problem [25, 26]. For instance, in high-income countries, obesity risk is elevated in children from families with low socioeconomic status, while risk is greater in the higher socioeconomic strata in lower-income countries [27]. Also, authoritative and permissive parenting styles have been associated with lower incidence of obesity, in contrast to an authoritarian style [28]. 

Through the lens of the Developmental Origins of Health and Disease, the seemingly unidentical twin maladies of early life under- and over-nutrition predispose individuals to identical, adverse health outcomes [29]. Despite extensive research on malnutrition and accumulating evidence of lifelong health implications, the pathophysiological pathways common to acute malnutrition (SAM) and obesity remain poorly elucidated [30]. This review uniquely juxtaposes these disorders, proposing that despite discrepant etiologies, early-life nutritional imbalances whether deficiency or excess, converge on common endocrine, immunological, epigenetic and gut-microbiome-related pathways and metabolic outcomes, with intergenerational implications.

## Methods

We searched for peer-reviewed, scientific papers, reports, and reviews using combinations of key terms such as child undernutrition, severe acute malnutrition (SAM), kwashiorkor, wasting, marasmus, pediatric obesity, endocrine alterations, immune dysfunction, epigenetics, DNA methylation, histone modifications, inflammation, and gut microbiome or microflora, metabolic outcomes, transgenerational effects and therapeutic approaches. Studies that met the following criteria were included – research conducted in pediatric populations, relevant animal models, documented mechanistic pathways relating to endocrine, immunological, epigenetic, and microbiome-related effects of child malnutrition, and long-term health outcomes. Studies focusing on adults or other non-child populations, those lacking primary data or molecular or metabolic focus were excluded. We synthesized findings to highlight similarities and contrasts between acute undernutrition and obesity across these domains.

### Metabolic Changes in Acute Undernutrition and Obesity

Despite being on distinctive ends of the nutrition spectrum, acute undernutrition and obesity in childhood both trigger devastating and often intersecting endocrine maladaptations. These maladjustments, summarized in Table 1, include β-cell dysfunction, insulin resistance, and altered growth-related hormone dynamics, which disrupt metabolic homeostasis and impair child growth.

## Endocrine Adaptations To Acute Undernutrition

Protein and energy deprivation in acute undernutrition alters key endocrine regulators of metabolism [31]. In severe PEM, there is a marked decline in the hepatic synthesis of transport proteins including thyroxine-binding globulin (TBG), thyroxine-binding pre-albumin (TBPA), and albumin [32]. This precipitates reductions in total circulating thyroid hormones such as thyroxine (T4) and triiodothyronine (T3) [32, 33]. Because most T3 is primarily produced from peripheral 5′-deiodinase–mediated conversion of T4, decreased conversion and increased production of reverse T3 further lowers free T4 and T3 levels [32, 34–36] Elevated inflammatory cytokines including IL-1, IL-6, TNF-α also suppress the release of thyroid-releasing hormone (TRH) and thyroid-stimulating hormone (TSH), and inhibit type 1 deiodinase activity, compounding the reduction in active T3 [35, 37]. These changes in thyroid hormone levels, collectively known as the “sick euthyroid syndrome,” represent an adaptive metabolic response aimed at conserving energy and limiting protein catabolism [35, 37]. However, these adaptive endocrine changes, when prolonged, are associated with a reduced metabolic rate and impaired growth in severely malnourished children [38–40] Recent studies report significantly lower free T3, free T4, TSH and serum albumin levels in SAM as compared to moderate acute malnutrition (MAM), with strong, positive correlations between albumin and thyroid hormones [32, 34, 41] Nonetheless, findings on TSH remain inconsistent, as some studies showed no differences between SAM and MAM [32, 33, 36] while others reported significantly higher [42] or lower [41, 43] levels in SAM.

Insulin-like growth factor-I (IGF-1) and insulin are also lowered to conserve energy and protein [44]. Interestingly, in NESAM, insulin secretion is paradoxically reduced, yet insulin resistance occurs [45, 46]. Possible mechanisms include β-cell dysfunction caused by decreased β-cell mass, oxidative and endoplasmic reticulum stress, impaired insulin synthesis possibly involving mTOR downregulation as shown in experimental models, disrupted amino acid–sensing pathways, and pancreatic atrophy, fibrosis, and apoptosis [45–48] These alterations reflect a coordinated metabolic adaptation to sustain glucose availability and vital organ function under nutrient deprivation [49]. While earlier animal studies reported hepatic insulin and glucagon resistance in chronic malnutrition, a more recent study with a small, pediatric sample found no evidence of peripheral or hepatic insulin resistance in ESAM [47]. 

Furthermore, acute malnutrition elevates Growth Hormone (GH) and cortisol which promote glycogenolysis, lipolysis and gluconeogenesis, causing wasting of skeletal muscles as amino acids and intermediate metabolites are used up [51]. This increased cortisol has been associated with stress induced by the activation of the hypothalamus-pituitary-adrenal axis, as well as by hypoleptinemia reported in experimental models [44, 52]. GH facilitates growth via IGF-dependent pathways (hepatic secretion of IGF-1, and proliferation and maturation of IGF-sensitive, epiphyseal chondrocytes) and other direct, non-IGF-dependent mechanisms [53–55] In SAM, despite raised concentrations of GH, circulating IGF-1 levels remain low, possibly indicating a resistance to GH-mediated production of IGF-1 [44]. This fall in IGF-1 generation induced by GH-resistance promotes lipid catabolism, oxidation of fatty acids and ketogenesis, an adaptive response to energy deprivation that deprioritizes linear growth to promote euglycemia and ensure survival [44]. 

In addition to protein-energy malnutrition, specific micronutrient deficiencies (e.g., zinc, magnesium, iodine and Vitamin B6) in SAM, also contribute to growth faltering by hampering the insulin-like growth factor (IGF) complex, which controls healthy growth and repair of tissues [56, 57]. Energy deficits in SAM reduce growth-regulating hormones including leptin, insulin, IGF-1, and increase glucocorticoids and IGF binding proteins which lower IGF-1 [58]. Low IGF-1, which is sensitive to nutrient intake and associated with organ growth/differentiation, slows down growth in undernourished children [59, 60]. IGF-1 can thus serve as a biomarker of dietary protein quality and nutritional status in children with altered growth patterns [57]. 

## Endocrine Dysregulation in Obesity

Unlike acute malnutrition, children with obesity generally exhibit normal, moderately [61] or abnormally [62] elevated serum TSH concentrations compared with their normal-weight counterparts. Free T4 (FT4) levels tend to be normal or slightly reduced [62], while total T4 may be raised [63] or unaltered [64, 65]. Additionally, total T3 concentrations may be mildly increased [66], suggesting an adaptive enhancement of peripheral T4-to-T3 conversion to attenuate fat accumulation via elevated energy expenditure [67]. These findings indicate a subtle alteration of the hypothalamic–pituitary–thyroid axis in pediatric obesity [65]. Nonetheless, raised thyroid hormones have been associated with cardiometabolic disease risk and Metabolic Dysfunction–Associated Fatty Liver Disease [68, 69]. Despite inconclusive evidence on the link between IGF-1 and pediatric obesity, some studies have shown that increased circulating IGF-1 in pre-pubertal children with obesity may be associated with increased nutritional status or dysregulation of other hormones [70–73] Elevated IGF-1, along with hyperinsulinemia, increased leptin, thyroid hormones, and sex steroids, may contribute to accelerated skeletal maturation in children with obesity [74]. This advancement in bone age can result in a slightly shorter adult height, although several studies report comparable final stature between children with and without obesity [75, 76] Despite acting through opposite biological pathways, growth attenuation is also observed in pediatric undernutrition, though typically to a much greater extent. Over-secretion of insulin may be triggered by the hyperreactivity of β-cells to overconsumption of insulinogenic nutrients like carbohydrates and lipids, to compensate for decreased sensitivity to the hormone, in a vicious cycle that further increases weight gain [77–78] Thus, while undernutrition is characterized by insufficient insulin secretion, β-cell failure and hepatic insulin resistance, obesity induces insulin resistance in muscle, liver and adipose tissue through β-cell overstimulation and chronic hyperinsulinemia [45–48, 50, 77, 78] Despite these opposite mechanisms, both conditions converge on β-cell dysfunction and disrupted insulin signaling, leading to impaired glucose regulation.

Although cortisol production and bioavailability are increased in both SAM and obesity, the underlying mechanisms diverge sharply and the physiological outcomes are disparate – muscle wasting in the former and exacerbated weight gain in the latter [79, 80]. In obesity, cortisol dysregulation may be linked with metabolic alterations in adipose and liver tissues [81]. Expansion of adipose tissue increases expression of 11β-hydroxysteroid dehydrogenase type 1 (11β-HSD1), which converts inactive cortisone to active cortisol, thereby amplifying cortisol exposure in local tissues [82, 83]. Also, hyperactivation of the hypothalamus-pituitary-adrenal axis, altered metabolic clearance of cortisol, coupled with changes in corticosteroid-binding globulin and corticosteroid receptor sensitivity may contribute to increasing cortisol availability [79, 84–89] Together, these processes create a state of tissue-specific cortisol excess, promoting visceral adiposity, insulin resistance, and metabolic dysfunction [79, 84–89] It is however noteworthy that the relationship between cortisol levels and obesity are inconsistent, as some investigators have documented positive, null, or even inverse associations, likely reflecting complexities in glucocorticoid production, variable cortisol clearance, and metabolic regulation [84, 90–95].

Increased leptin concentrations associated with increased body fat mass have been documented in childhood obesity, and leptin has also been shown to respond to weight loss, thus suggesting a possible biomarker role for leptin in obesity and obesity treatments [96, 97]. In contrast to acute undernutrition, hyperinsulinemia and high body fat composition in obese children significantly decrease GH concentrations (~ 50%) [65]. Although both conditions involve dysregulation of the GH-IGF axis, their growth outcomes differ. Acute undernutrition is associated with markedly diminished adult height [31]. In contrast, obese children are often taller than normal weight peers in pre-puberty; however, earlier onset of puberty, reduced pubertal height gain, and accelerated epiphyseal maturation typically may predispose them to final heights that are similar to or slightly below genetic growth potential [98–101].

**Table 1:**
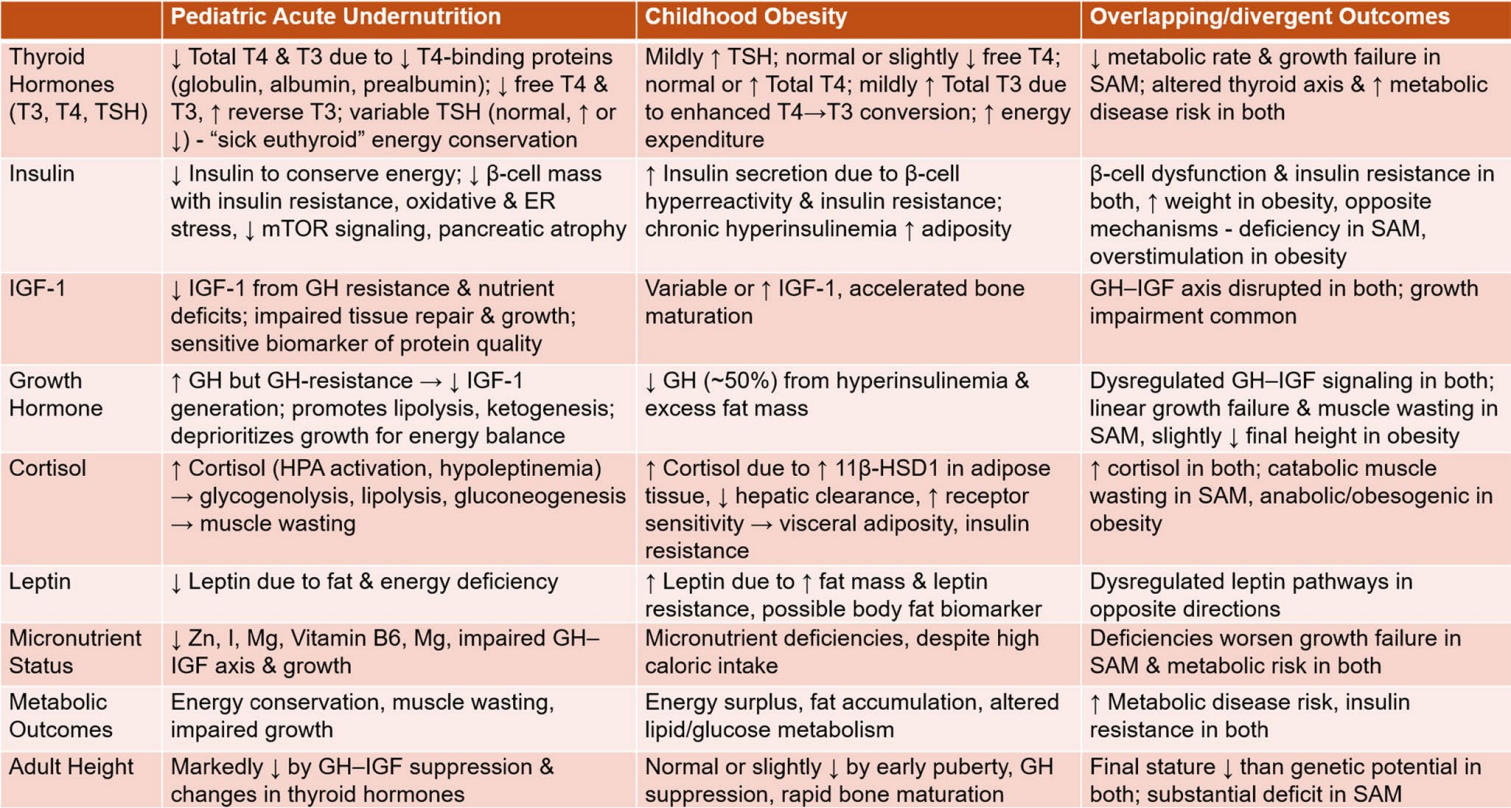
Metabolic and endocrine adaptations to pediatric acute undernutrition vs. obesity

## Molecular Changes in Under- and Overnutrition

**Fig. 1:**
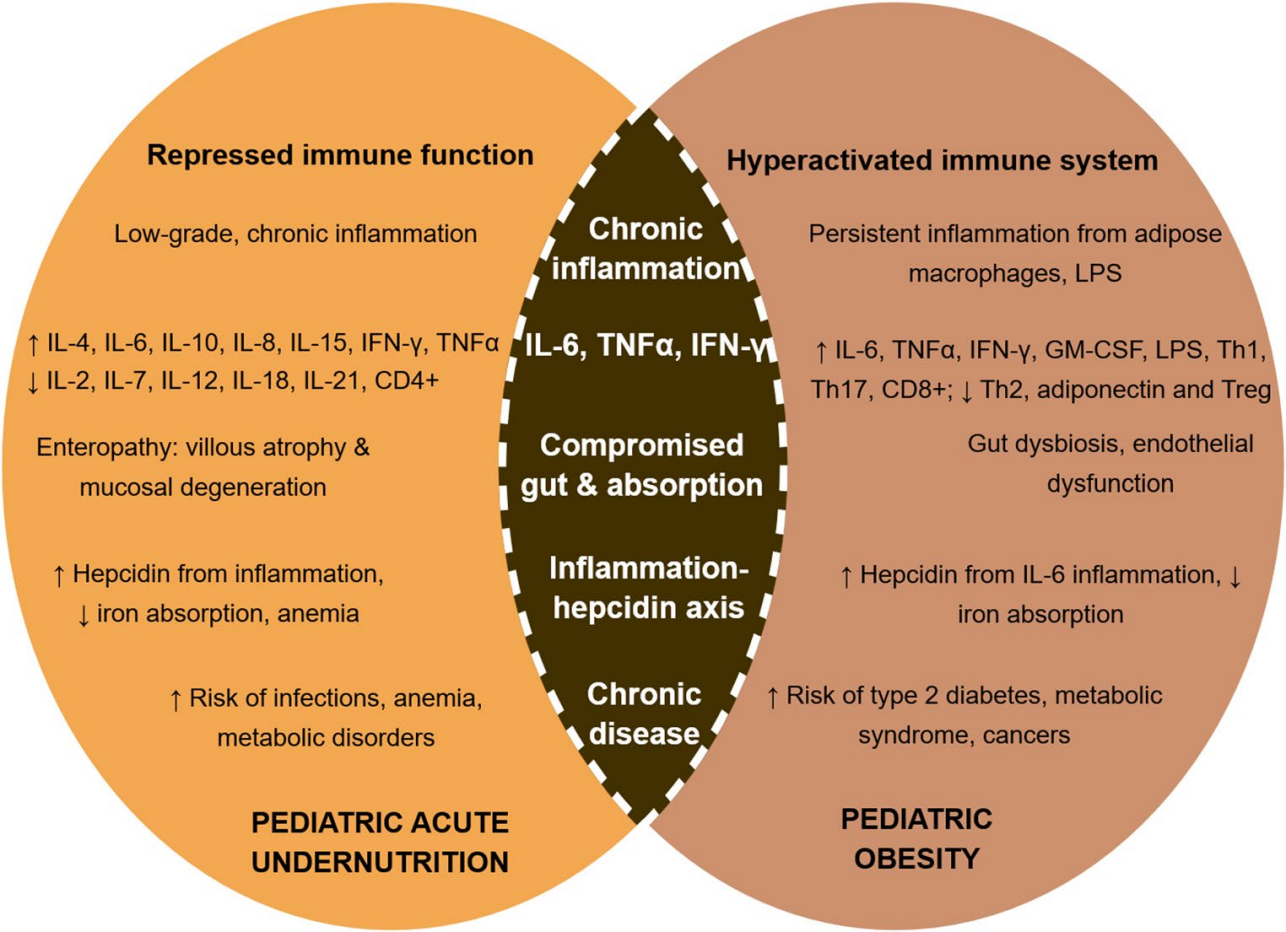
Immune dysfunction in pediatric acute undernutrition vs. obesity

Despite divergent nutritional conditions, both childhood acute undernutrition and obesity disrupt immune function in ways that overlap through chronic inflammation. In childhood SAM, immune function is repressed, aggravated by micronutrient deficiencies and resulting in sustained, low-grade inflammation, long-term metabolic damage and increased odds of infections and mortality [51]. Conversely, pediatric obesity is characterized by immune hyperactivation, causing a similar cascade of inflammation-mediated impairments in the absorption and utilization of key nutrients, particularly iron. These disturbances are driven by IL-6–induced hepcidin upregulation, increased nutrient demands, and systemic metabolic dysregulation [102, 103]. Together, these immune disruptions, summarized in Fig. 1, underlie a lifelong susceptibility to chronic metabolic diseases.

## Immune Dysfunction in Acute Undernutrition: Driver of Acute and Chronic Disease Outcomes

Immune dysfunction in SAM predisposes children to acute infections including enteric and respiratory illnesses, measles and malaria, while survivors experience growth failure and long-term metabolic disorders [104–106] Both acute undernutrition and obesity precipitate micronutrient insufficiency, which exacerbates the severity of these conditions in mutually reinforcing cycles [51, 107–109] Enteropathy, characterized by gut mucosal degeneration, villous atrophy and impaired gut structure, occurs through a cascade of nutrient deficiency-induced biochemical, physiological and histological changes [108, 109]. These distortions depress absorption of key nutrients (folate, zinc, protein and Vitamin A) crucial to cell division and proliferation, aggravated by rapid DNA replication and cell turnover in the gut mucosa [106]. Disturbances in the intestinal endothelium, lymphoid atrophy, gut dysbiosis, and elevated inflammatory metabolites (TNF, specific pteridines, IL-1, IL-6 and IL-12, fecal calgranulin and myeloperoxidase) further compromise intestinal function [51, 106, 110–112] SAM-induced inflammation further inhibits micronutrient uptake, creating a downward spiral in SAM severity and increased morbidity and mortality [104, 113]. For instance, high pro-inflammatory cytokines in SAM upregulate hepcidin expression, diminishing iron bioavailability and erythropoiesis and precipitating anemia [113]. 

In comparison with controls, children with acute undernutrition exhibit higher levels of chemokines and cytokines: IL-4 and IL-10 required for Th2 activity and IL-8, IL-15, IFN-γ, GM-CSF, and TNFα which stimulate Class I cytokine receptors and G protein-coupled receptors to transduce pro-inflammatory signals [112, 114, 115]. Conversely, decreased cytokines essential for Th1 cell differentiation and function (IL-2, IL-7, IL-12, IL-18, and IL-21) and antigen-experienced T cells like CD4^+^, CD45RO^+^ and effector T cells have been reported in hospitalized children with SAM [105, 116–118] All of these factors in tandem impair immune function and elevate susceptibility to infections in children with SAM, setting up a vicious cycle of malnutrition and infection which increases the severity and fatality of SAM [106, 117–120]. Additionally, despite the absence of pathogenic infections, acutely malnourished children exhibit elevated concentrations of IFN-γ, IL-6, and IL-1β, indicating persistent, low-grade systemic inflammation, which may play a crucial role in non-communicable disease conditions including diabetes, cardiometabolic disorders and other diseases of the metabolic syndrome [112, 120, 121]. 

### Immune Activation in Childhood Obesity: the Hidden Inflammatory Burden

Both acute undernutrition and obesity have convergent, long-term outcomes (chronic, metabolic diseases like insulin resistance, type 2 diabetes, cancers and others) and similar pathways through inflammation, with diverse specific mechanisms [2, 122]. Micronutrient deficiencies in childhood obesity result from complex causes including high-calorie diets low in micronutrients, malabsorption due to chronic inflammation and endothelial dysfunction, and increased hematopoietic nutrient requirement (iron) for high blood volume to accommodate increased adipose tissue [108, 123, 124]. Similar to acute undernutrition, iron deficiency is triggered in obese children by elevated levels of proinflammatory cytokines [108, 113]. These inflammatory metabolites (IL-6 and other proteins) raise circulating hepcidin, upregulate ferritin, preventing intestinal iron absorption, and promoting degradation and removal from the blood into the cells [108, 124]. Macrophages are the most abundant immune cell in adipose tissue, with higher numbers of macrophages coinciding with greater severity of obesity [122]. Obesity favors the classically activated, pro-inflammatory M1 type macrophages, T-lymphocytes and adipokines which increase inflammatory metabolites similar to those found in undernutrition, including GM-CSF, IFN-γ, TNFα, IL-6 and lipopolysaccharides (LPS) [122, 126]. Contrary to the reduced Th1 and increased Th2 activity in SAM, pro-inflammatory Th1, Th17, and CD8 + are more active in obesity compared to anti-inflammatory Th2, adiponectin and Treg [127]. 

## Epigenomic Effects of Nutritional Imbalances

**Fig. 2:**
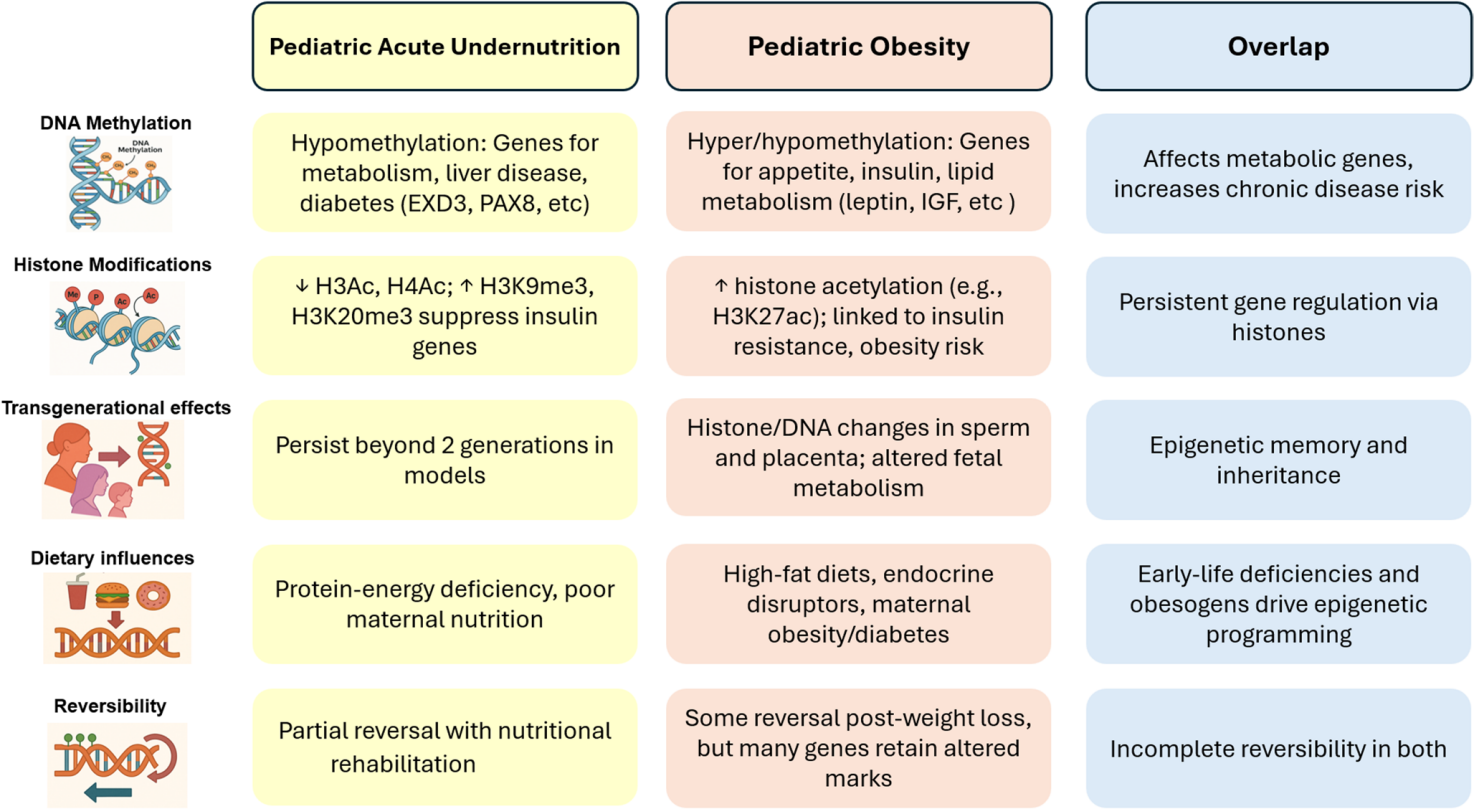
Epigenomic Effects of Nutritional Imbalances

Both acute child undernutrition and obesity alter gene expression, cell signaling, and nutrient utilization triggering extensive, epigenetic modifications, perturbing metabolism and escalating chronic disease risk [9, 128, 129]. These epigenetic mechanisms include differential DNA methylation, histone modifications, and miRNA expression that affect genes modulating energy metabolism, appetite control, and insulin signaling [121, 130, 131]. Differential methylation and histone modifications, summarized in Fig. 2, target distinct gene sets in both conditions, but metabolic and disease outcomes overlap. It is established that some epigenetic modifications may be partially reversible, yet there is increasing evidence of persistence of others into adulthood and across generations.

## Epigenetic Modifications and Outcomes in Pediatric Acute Undernutrition

### Differential DNA Methylation Signatures in SAM

Epigenetics (DNA methylation, post-translational histone modifications and post-transcriptional microRNA (miRNA) may contribute to the phenotypic divergence between ESAM and NESAM, predisposing survivors to increased metabolic disease risk later in life [9, 132]. In Malawi and Jamaica, children with ESAM exhibited significant hypomethylation in 99% of 877 differentially methylated CpG sites on genes related to fatty liver, obesity, diabetes and cardiometabolic disorders, in comparison with their counterparts with NESAM [9]. Similarly, in an epigenome-wide DNA methylation mapping of adults in Barbados previously exposed to postnatal acute malnutrition, a total of 303 genes regulating metabolism and obesity remained differentially methylated, with GNAS being the largest locus [132]. In Bangladeshi adults exposed to famine in early life, metastable epialleles, such as Vault RNA 2–1, PAX8, EXD3, PRDM9, ZFP57, and BOLA2, which are involved in abnormal glucose tolerance, were differentially methylated in comparison with controls [133]. In adult SAM survivors in Jamaica, a total of 133 CpG sites across 63 genes associated with body size and composition, as well as metabolic pathways involved in immunity, cardiovascular function, and glucose regulation were found to be differentially methylated [134]. These variations in DNA methylation may provide an explanation for the increased glucose intolerance, poorer beta cell function, and differences in cardiac anatomy and physiology in adults with a history of SAM [132, 133]. 

Protein deficiency in SAM disrupts many pathways in energy metabolism including the Tricarboxylic Acid Cycle, the Electron Transport Chain, and β-oxidation and perturbs gut microbiota, iron and amino acid metabolism [135, 136]. In a rat model simulating SAM conditions, ~ 24% of hepatic genes were differentially expressed with upregulation of *TRIB3* and *FGF21* (markers of disrupted hepatic signaling) and long-lasting steatosis was attributed to peroxisome loss via transcription-mediated pexophagy [137]. Also, a different rat model of a low protein diet reported upregulation of genes involved in de novo lipogenesis (SREBP1C, FASN, ACC, and SCD1), and downregulation of genes associated with mitochondrial dysfunction (PGC1Α, TIM23, AND TFAM), contributing to liver steatosis and fibrosis [136]. In children with ESAM, differential methylation has been observed at loci linked with metabolic dysfunction–associated fatty liver disease (MAFLD), including NDUFS1 (involved in mitochondrial complex I function), PHLDA1 (associated with adipogenesis), NFE2L1 (essential for hepatic proteasome function), and SLC2A4 (encoding an insulin-sensitive glucose transporter) [9, 137–140]. Furthermore, increased 16-carbon monounsaturated fatty acids, and 16–18-carbon saturated fatty acids and lysophosphatidylcholines (LPCs) have been shown in children affected by SAM [141]. LPC activates the GPCR/JNK cascade and the Endoplasmic reticulum (ER) stress–CHOP–JNK axis, promoting hepatocyte apoptosis and contributing to the progression of steatosis through lipotoxic injury [142]. In summary, human SAM studies indicate that hepatic steatosis is associated with epigenetic dysregulation of metabolic and signaling pathways, whereas animal models reveal transcriptional reprogramming involving peroxisome loss, stress responses, and disrupted mitochondrial function. Hepatic steatosis is further associated with dyslipidemia, increased inflammation and higher incidence of insulin resistance and atherosclerosis, again denoting a link between SAM and chronic metabolic diseases [104, 137]. 

The effects of molecular reprogramming associated with undernutrition are extensive and impact glucose metabolism. Recently, a novel diabetes classification, Type 5 diabetes, has been proposed by the International Diabetes Federation, to describe a subtype emerging in lean, young individuals exposed to undernutrition during fetal and early postnatal life [143]. Unlike classical type 2 diabetes marked by insulin resistance, this phenotype, estimated to affect 25 million people in LMICs, is characterized by severe pancreatic β-cell dysfunction with substantial deficits in insulin secretion, yet insulin sensitivity is preserved, and autoimmunity or ketoacidosis are absent [144]. It is believed that protein and micronutrient deficiencies in early childhood impair pancreatic development and β-cell function, ultimately reducing insulin secretion through pathways involving oxidative stress-induced β-cell apoptosis and epigenetic alterations in key transcriptional regulators of insulin gene expression, such as PDX1 and MafA [144–146] The recognition of Type 5 diabetes highlights undernutrition as an independent mechanistic driver of metabolic disease and reinforcing the need for nutritional and context-specific therapeutic strategies in affected populations.

### Obesity-Associated DNA Methylation Profiles in Early Life

Although obesity is a transgenerational health issue, recent GWAS studies suggest that genetic variation accounts for 40–50% of the variance in obesity rates [146]. Changes in the food and work environment may contribute to obesity through epigenetic modifications [148]. Previous studies have shown an association between obesity and DNA methylation of leptin and adiponectin gene promoters, two hormones that work within adipose tissue and are involved in energy balance regulation and fat storage [149, 150]. Other genes related to appetite regulation and obesity, including NPY, POMC, and IGF are also regulated by both DNA methylation and histone modification [151]. 

There is evidence that the obesogenic environment also triggers epigenetic modifications that increase risk of obesity. Endocrine disruptors, like those found in plastics, are associated with increased risk of metabolic disorders, including obesity [152]. Dietary factors also likely contribute to epigenetic modifications. Studies have shown both widespread reductions in DNA methylation and specific reductions in methylation of leptin and melanocortin 4 receptor after short- and long-term high fat intake [153, 154]. Intake of a high fat diet during pregnancy can also impact epigenetic regulation in the fetus [155]. Gestational diabetes can also lead to epigenetic modifications, impacting the growth of the fetus and birth weight [156, 157]. 

In a recent EWAS study, 792 genes were hypermethylated and 2341 were hypomethylated in children with overweight/obesity as compared to those with normal weight, with the genes PCDHGA4, BRD2 and PTPRN2 involved in regulating dysglycemia and the hypothalamic appetite network, obesity and type 1 diabetes having the highest number of differentially methylated sites [158–160] Another study comparing obese vs. normal weight children showed 241 differentially methylated sites in genes involved in fat, carbohydrate and protein metabolism, as well as metabolic and disease conditions including insulin dysregulation, type II diabetes, and cardiovascular diseases [161]. Although the specific genes and pathways may vary, differential DNA methylation in pediatric under- and overnutrition affect genes regulating similar metabolic processes and result in similar disease outcomes.

Epigenetic changes induced by pediatric obesity may be transgenerational. At the 10-year follow-up mark of an investigation of cardiometabolic outcomes of pediatric obesity, 30 of 376 cardiomyopathy-associated differentially methylated sites at baseline persisted, with 11 being linked to cardiac hypertrophy in adulthood [162]. Differential DNA methylation modified expression at specific sites in the KAZN, TDH and SLC17A9 genes, associated with alterations in heart structure [162]. Furthermore, epigenetic memories have been shown to persist in human adipose tissue, denoting incomplete recovery of metabolic function, and may explain the accelerated rebound obesity in individuals with subsequent exposure to obesogenic lifestyles, after substantial weight loss [163]. For instance, numerous, differentially expressed metabolism-regulating genes in obesity remained remarkably downregulated after significant weight reduction (including IGF-1, LPIN1, IDH1, PDE3A, DUSP1, GPx3, and Glutamine Synthetase genes), while fibrosis- and apoptosis-related pathways linked with TGF-β were upregulated [164]. Also, hypomethylation of GRP78, *FTO* and *TNF* and PDGFR-β, and hypermethylation of leptin, PPARG, LXRα, Lipin 1 and GRP78 in mothers have been shown to predispose offspring to obesity, disruption of hepatic lipid metabolism and other cardiometabolic outcomes [164]. These studies highlight the long-term individual and transgenerational, epigenetic impact of pediatric obesity, which are similar to pediatric undernutrition.

### Histone Modifications in SAM

Through epigenetic pathways, histone acetylases (HATs) and histone deacetylases (HDACs) promote and repress gene expression, respectively, by opening or closing the chromatin structure [165]. Although epigenetic modifications related to pediatric undernutrition are increasingly being studied, most investigators have focused on DNA methylation changes [9, 166–168] Recently, some researchers have begun to explore histone modifications in chronic child undernutrition (stunting), but there is sparse information relating to these epigenetic modifications in acute child undernutrition [169–171] Nonetheless, in a rat model of protein-energy deprivation over 50 generations, which simulates conditions in low-income countries, Hardikar et al. showed an upregulation of the enzyme Histone-lysine N-methyltransferase (KMT1A) in the pancreatic Islets [172]. KMT1A has been shown to suppress insulin-2 gene transcription by the addition of three methyl groups to Histone H3 lysine-9 methylation (H3K9me) [172]. Even after two generations of dietary recuperation, the suppression of gene transcription persisted [172]. Further epigenetic evaluation revealed reductions in three histones (H3Ac, H4Ac, H3K4me3) that transcriptionally activate insulin-2 gene promoters and significant elevation in two histones (H3K9me3, H3K20me3) that suppress/silence these promoters [172]. These epigenetic changes were associated with excessive visceral fat accumulation and disorders of the metabolic syndrome including insulin resistance/hyperinsulinemia, high circulating homocysteine, endotoxemia, elevated leptin concentrations, lower adiponectin and a higher risk of diabetes [172]. Although nutritional rehabilitation over two generations did not completely reverse the epigenetic remodeling, it increased the activating histone marks and reduced the repressive ones [172]. It is pertinent to note that while histone- and microRNA-mediated transcriptional regulation are emerging as important epigenetic mechanisms in nutritional programming, evidence in SAM remains limited and is mostly derived from animal models, underscoring a critical gap that warrants further investigation.

### Histone-Mediated Epigenetic Changes in Pediatric Obesity

Despite the growing interest in epigenetics in childhood obesity, few studies have examined histone modifications and their metabolic outcomes. Higher histone acetylation has been reported in children with obesity, corresponding with higher circulating insulin, insulin resistance and the pro-inflammatory metabolite TNF-α, as well as a downregulation of the expression of the anti-inflammatory *SIRT1* gene [173]. Additionally, histone acetylation was positively correlated with weight, Body Mass Index, waist circumference and waist-to-hip ratio, suggesting a possible role in body weight and fat regulation [173]. 

The interaction between DNA methylation and histone modifications, two major epigenetic mechanisms, also has adverse effects on child health outcomes. In placental tissues obtained from offspring of obese parents, DNA hypermethylation dysregulated the expression of histone deacetylase 4, (HDAC4), which modulates fat cell expansion, promotes energy utilization and protects from excessive weight gain [174–177] This dysregulation could elevate metabolic disease risk in offspring later in life [174]. 

The effects of histone modifications in obesity have also been shown to be long-term and transgenerational. For instance, in obese mice fed a high-fat diet, paternal sperm showed altered histone marks (H3K4me3, or trimethylated histone H3) in genes regulating metabolism [178]. Corresponding histone changes were found in the placentas of their embryos, along with disrupted placental function and development, suggesting possible metabolic disease programming in offspring [178]. Although some studies have shown that obesity-induced histone alterations could be reversed, the persistence of some modifications have been established. For instance, in a vertebrate model of early life obesogenic diets, histone modifications (H3K27ac and H3K9me3) related to hepatic steatosis were mostly reversed after eight weeks of a normal diet, but 16 genomic loci retained a permanently altered histone mark [179]. Another study also found that in mice fed a high fat diet, 44 of 680 differentially expressed, metabolism-regulating genes remained upregulated even after dietary normalization, and the histone modification (H3K9me2) was associated with less accessible chromatin, potentially contributing to long-term risk of chronic, metabolic diseases [180]. These findings reveal similar histone modifications and their long-term or transgenerational, epigenetic outcomes in childhood undernutrition and obesity.

## Changes in Gut Microflora Induced by Acute Undernutrition and Obesity

### Dysbiosis in Gut Microbiome in Acute Undernutrition

**Fig. 3:**
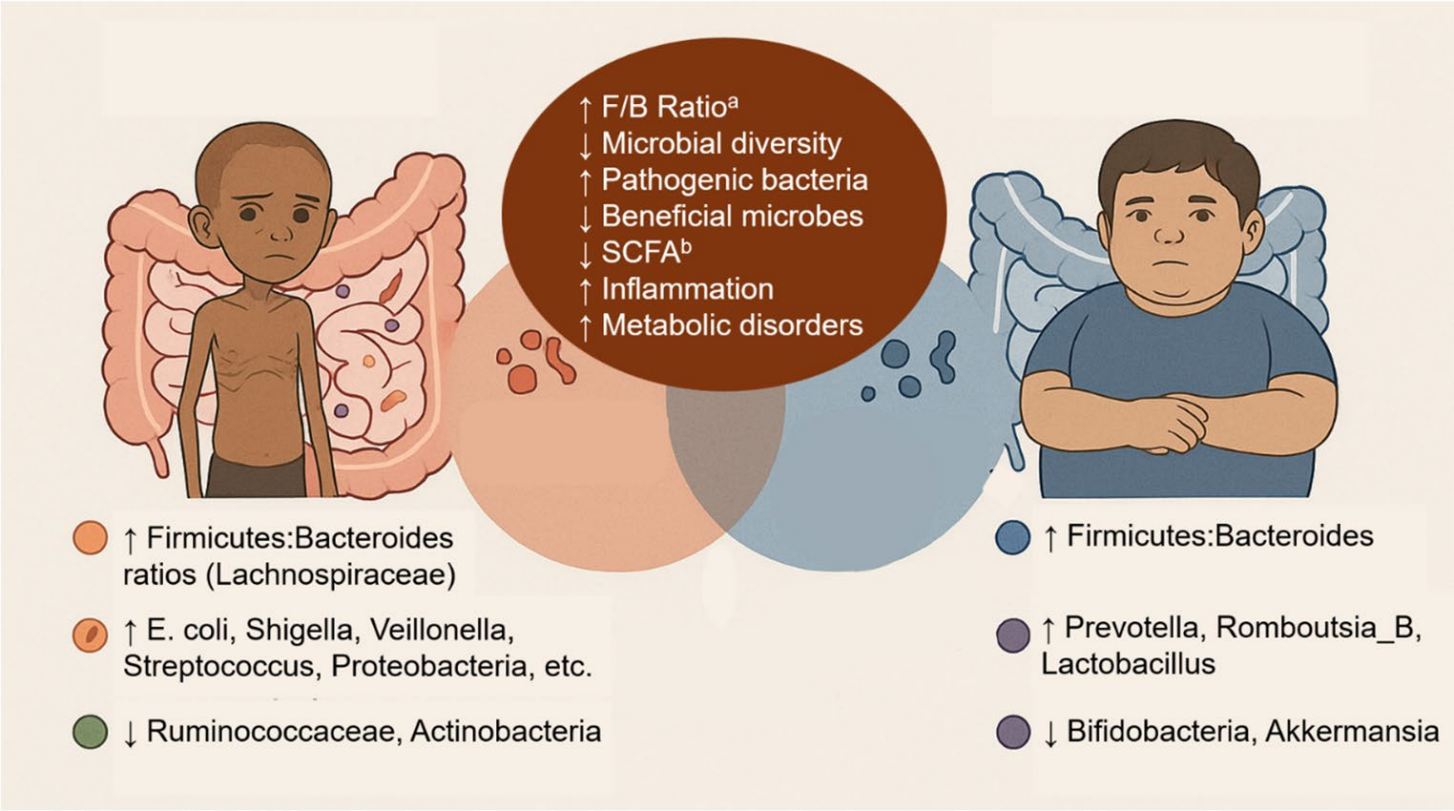
Changes in gut microflora in pediatric acute undernutrition vs. obesity. ^a^ F/B ratio = Firmucutes/Bacteroidetes ratio, ^b^ SCFA = Short chain fatty acids

Severe acute malnutrition is accompanied by disruption in gut microbial pathways [181, 182]. Fecal microflora in acutely malnourished children with SAM was less mature (with lower diversity) than that of healthy children [183]. These changes in host microbiota in SAM, summarized in Fig. 3, are probably responsible for the dysbiosis-mediated, abnormal regulation of intestinal immune function that is linked to an increased risk of infection [184]. In undernourished children in Indonesia and Mexico, studies have shown increased Firmicutes: Bacteroides ratios, particularly *Lachnospiraceae.* [183, 185] While higher proportions of Firmicutes to Bacteroides have been associated with higher risks of obesity, type 2 diabetes and breast cancer, the evidence remains inconclusive, as findings vary widely due to methodological biases, interindividual differences and lifestyle factors [185–193] Thus, the Firmicutes: Bacteroidetes ratio is not considered a reliable or reproducible biomarker of nutritional status [191]. Furthermore, elevations in the populations of pathogenic microbiota, including *Escherichia coli*, *Shigella*, *Streptococcus*, *Proteobacteria*, *Fusobacteria*,* Enterococcus fecalis*,* Staphylococcus aureus* and *Veillonella* have been documented in undernutrition, while beneficial bacteria Ruminococcaceae, and Actinobacteria were reduced [181, 194–197].

Short-chain fatty acids (SFCAs) like acetate, butyrate and propionate, derived from host microbial fermentation of non-digestible carbohydrates, transduce signals through GPCR to promote anti-inflammatory cytokines (IL-10, IL-18) and Treg responses [198, 199] These SCFAs also suppress pro-inflammatory histone deacetylase (HDAC), NF-κB and nitric oxide production, thus diminishing oxidative stress-induced epithelial cell apoptosis, and exerting a protective effect on colonic and cardiac health [199]. SCFAs have been shown to regulate carbohydrate, lipid and overall energy metabolism, reduce inflammation and adiposity, modulate appetite and promote insulin sensitivity [200, 201] In a study in Indonesia, moderately undernourished children were shown to have lower concentrations of propionate and butyrate [202]. In addition to metabolic disorders, low concentrations of SCFAs have been linked with higher mortality in undernourished children [203]. 

### The Gut Microbiome and Obesity

There is strong evidence that the gut microbiome contributes to obesity. Studies in animal models show that genetically obese mice have a different microbiome composition than wildtype mice and that transfer of the obese mice microbiome into germ free mice leads to excess accumulation of adipose tissue [204, 205]. Colonization of germ free mice with human microflora has been shown to have the same effect [206]. Children may be especially vulnerable, as their microbiome is established at birth and highly variable depending on the mode of birth and the infant feeding approaches used by the mother [207]. The early life gut microbiome has been shown to be associated with childhood overweight and obesity [208]. 

Many studies have suggested that an increase in *Firmicutes* and a decrease in *Bacteroidetes* are most critical for obesity risk [209]. Yet, there is paucity of data from investigations of the gut microbiome in children, and the limited results linking these microbes to obesity in this population seem inconsistent [209, 210]. Nonetheless, similar to reports in acute undernutrition, Ma et al. have documented greater ratios of Firmicutes/Bacteroidetes (F/B) in children with obesity as compared to normal weight peers [211]. Also, obese children have been found to have reduced levels of *bifidobacterium*, Oscillospira, and Akkermansia and butyrate-producing microbiota, as well as elevated levels of *Prevotella* [210, 212]. It should be noted, however, that increased *Prevotella* levels have been linked with both beneficial and detrimental metabolic health outcomes, possibly related with strain-level heterogeneity and metabolic versatility [213]. Also, *Prevotella* abundance has been documented in diverse dietary contexts, including both high-fiber and Western diets, likely arising from genomic and functional variability among strains [213, 214]. Thus, the association of *Prevotella* with childhood obesity may reflect context-specific interactions between host metabolism, dietary composition, and microbial functional capacity.

Insufficient dietary substrates for microbial synthesis of short chain fatty acids including butyrate, may promote obesity [215]. A randomized controlled trial that administered butyrate supplements to obese children resulted in significant reductions in BMI, waist circumference, insulin concentrations, ghrelin, IL-6 and increased insulin sensitivity [200]. Although promising, these preliminary findings should be interpreted cautiously given the study’s small sample size and short duration. The composition of the gut microbiome is sensitive to dietary factors. High fat and high carbohydrate diets lead to gut microbiome dysbiosis and metabolic disruption [216]. In a mouse model of diet-induced obesity, diminished Akkermansia and Intestinimonas correlated with heavier organ weights and elevated blood glucose, low density lipoprotein, and inflammation, while higher levels of Lactobacillus and Romboutsia_B correlated with higher blood lipid levels and inflammatory biomarkers [217]. Conversely, healthier diets, such as the Mediterranean Diet, are associated with a healthy gut microbiome composition [218]. In sum, gastrointestinal dysbiosis disrupts immune function and metabolic homeostasis through divergent pathways in acute child undernutrition and obesity, with grave and enduring health consequences which may be partially remedied through dietary interventions.

A key mechanism linking the gut microbiome to obesity appears to be the enhanced harvesting of energy from food in the obese microbiome [204]. Additional mechanisms have been proposed for the relationship between the gut microbiome and obesity including bile acid signaling, alterations in short-chain fatty acid (SCFA) production, and inflammation associated with enteric-derived lipopolysaccharides (LPS). Gut microbiome-produced bile acids influence glucose and lipid metabolism, energy balance, and inflammation via FXR and TGR5 receptor signaling, thus, alterations in bile acid profiles may disrupt these pathways and contribute to metabolic disease [219]. Aberrant SCFA production can promote adiposity through GPR41- and GPR43-mediated disruptions in adipogenesis, lipolysis, and gut-brain signaling [220]. Furthermore, chronic, low-grade inflammation triggered by LPS-induced activation of TLR4-NF-κB impairs insulin signaling, enhances lipogenesis and adipocyte hypertrophy, promoting lipid accumulation and metabolic dysregulation [221]. 

## Long-term Outcomes of Childhood Malnutrition and Therapeutic Solutions

### Persistent Metabolic Consequences of Acute Undernutrition

Despite limited empirical evidence of the long-term health impacts of pediatric undernutrition, modified phenotypes and pathophysiology similar to those observed in obesity (higher cardiometabolic disease risk and significantly higher odds of poor health) have been shown to persist in adult survivors of SAM [10, 222–224]. Nonetheless, contrasting findings of non-significant cardiometabolic risks among adolescent SAM survivors have been documented, despite shorter height attainment during catch-up growth [225]. These discrepant results may be indicative that nutrition rehabilitation may mitigate the short-term effects of acute undernutrition in childhood, but may not completely alleviate the associated chronic disease risks and therefore necessitate further investigation. Overall, the evidence remains mixed, with stronger associations observed in adults than in adolescents.

### Chronic Impacts of Pediatric Obesity

Despite extensive studies of comorbidities in childhood obesity, only few, robust, longitudinal investigations have explored the metabolic and molecular underpinnings in adulthood, possibly due to the lengthy follow-up and extensive resources required [226–229] Relative risks ranging from 1.7 to 5.4 for type 2 diabetes, hypertension, dyslipidemia and cardiovascular diseases, and a 16.6% increased risk of multi-morbidities have been reported in adults with persistent obesity from childhood [230–232]. Also, 30- to 40-year follow-up data from the International Childhood Cardiovascular Cohort Consortium have revealed consistent links between childhood obesity and early mortality from cardiovascular diseases, cancers, abnormal lipid metabolism and diabetes in adulthood [233–236] Nonetheless, the seven cohorts in this consortium reside in high-income countries (US, Australia and Finland), thus, critical data from middle- and lower-income countries are lacking [237]. Given the rapid rise in obesity within LMICs, often coexisting with persistent early-life undernutrition as part of the dual burden of malnutrition, there is an urgent need for longitudinal studies that elucidate how these intersecting nutritional exposures shape long-term cardiometabolic risk in these contexts.

### Therapeutic Approaches and their Consequences in Acute Undernutrition

Ready-to-use therapeutic foods (RUTF) are the current method of choice for the treatment of SAM without complications, because they are effective, amenable for use outside of clinical settings, and do not need water for reconstitution [238]. This strategy has successfully improved the prognosis and survival of millions of children with SAM in the developing world, leading to a surge in the demand for the products [238]. RUTF, also known as lipid nutrient supplements, are calorie-dense, high-fat and high-sugar, nutty, paste-based foods, with adequate protein and micronutrients to promote rapid recovery from SAM [240]. Habitual inclusion of RUTF in young children’s diets may shape taste preferences toward sweeter or more energy-dense foods; however, evidence for lasting metabolic or epigenetic effects is limited and largely theoretical [238]. It is not clear if RUTF supplementation could be obesogenic in the long-term, as there is paucity of information on the long-term effects on SAM survivors.

### Interventions for Childhood Obesity and their long-term Implications

Current therapeutic options for severe obesity in childhood include lifestyle modifications (preferred approach), drug therapy, and surgical procedures [227]. In Sweden, risks of type 2 diabetes and dyslipidemia were reduced by 48% and 69%, respectively, in children who experienced resolution of obesity after varying treatments [240]. Several studies have reported short- (≤ 3 years) or medium-term (4–9 years) outcomes of combined strategies to reduce pediatric obesity, but only few have investigated long-term (> 10 years) impacts [241–244] For instance, a 4-year follow-up after a 12-month, integrated weight loss program substantially improved weight outcomes and diastolic and systolic blood pressure, particularly in children who participated around the age of 6 years [243, 245]. A recent systematic review has documented modest to substantial improvements in weight status and cardiometabolic outcomes, from behavioral and pharmacotherapeutic interventions for pediatric obesity [244]. However, most of these findings are based on short- to medium-term follow-up, and the durability of benefits over longer periods (> 10 years) remains unknown, particularly for lifestyle-based approaches [245]. Emerging evidence also points to favorable longitudinal metabolic effects of surgical weight treatment in adolescents, but long-term outcomes of lifestyle interventions, and studies in younger children, are still sparse.

## Global Implications, Limitations and Future Research Directions

This narrative review has provided a detailed account of the intersecting characteristics of the apparently discordant twin conditions of pediatric acute undernutrition and obesity. Taken together, both syndromes affect 77.8 million children globally, approximately 12% of the world’s 654 million children (0–5 years) [5, 246]. If left unaddressed, this heavy burden of child malnutrition portends catastrophic demographic, health and economic challenges to the collective future of the world.

This review has shown that as much as nutritional rehabilitation partially resolves some of the metabolic and molecular outcomes of child undernourishment and obesity, some of the damage is permanent. This creates a domino effect of susceptibility to debilitating chronic ailments in millions of children and their future offspring. The irreversibility of these effects thus necessitates a systems-thinking approach to proactive prevention of child malnutrition. Global determinants such as poverty, socioeconomic and health disparities, climate-induced crop failures, extensive ultra-processed food supply chains and aggressive marketing, structural environments limiting physical activities and access to healthy foods, urban and rural food deserts, unhealthy lifestyle practices, limited nutrition knowledge, and unfavorable beliefs, attitudes and behavioral dispositions contribute to child malnutrition in all its forms. Addressing these systemic drivers is as pertinent as understanding the underlying biological mechanisms.

To address the dual burden of malnutrition, policies must align with biological insights into how structural drivers shape pathways to obesity and undernutrition. Socioeconomic inequities and climate change can reduce access to nutritious foods, promote sedentary behavior, and heighten exposure to toxins, impairing the gut microbiome, epigenome, and metabolism. Similarly, habitual consumption of ultra-processed foods can induce gut dysbiosis and epigenetic changes in metabolic and inflammatory genes, disrupting metabolic homeostasis. Effective responses from governments and all stakeholders therefore need to transcend last-mile interventions, to encompass nutrition-sensitive and nutrition-specific solutions that address basic, underlying and immediate causes of child malnutrition. These may include fiscal and regulatory measures like taxing nutrient-poor, unhealthy foods, and subsidizing nutrient-dense staples, fresh fruits and vegetables and implementing clear front-of-pack labeling. Urgent action is also needed to restrict marketing of high-fat, high-sugar products to children and to promote access and demand for healthier options that incentivize industry reformulation. Governments can further provide free or insurance-covered caregiver education on infant and young child feeding, home gardening, and meal preparation, while fostering built environments that enable physical activity. Agricultural and environmental policies should likewise advance climate-robust, sustainable food production systems including the use of resilient, drought-resistant crops, circular farming and other practices that support healthy diets and planetary health.

Despite increasing interest and emerging evidence of the underlying pathophysiology of pediatric malnutrition in all its forms, substantial knowledge gaps remain. While exploration of DNA methylation patterns in children with acute undernutrition and obesity has garnered significant attention, research on other layers of epigenetic regulation, such as histone modifications and microRNAs, remains scarce. This study provided limited discussion of histone modifications and did not discuss the involvement of microRNAs in child malnutrition because of shortage of comparable data in undernutrition.

Additionally, a remarkable proportion of current insights on epigenetics in child undernutrition stems from animal models, or focus on chronic undernutrition (e.g., stunting), rather than acute forms. Furthermore, geopolitical disparities obscure our understanding, as pediatric obesity research mostly emanates from high-income countries, while SAM-related data predominantly originates from low- and middle-income countries, thus insights from significant swaths of pediatric populations are left unexplored. Also, most mechanistic, epigenetic, endocrine, and microbiome studies to date have been conducted in high-income settings, while studies from LMICs are more observational or epidemiologic, limiting the generalizability of findings.

Rigorous, longitudinal studies should be designed in diverse geographical and demographic contexts to examine the short- and long-term metabolic, molecular and health impacts of undernutrition and excessive body weight in childhood. These investigations should identify, validate and main-stream biomarkers including IGF-1, leptin, and other metabolites, which are sensitive to nutrient status, with the aim of providing early signals of child nutritional status that are timelier than anthropometrics. Also, there is a dire need for longitudinal studies to elucidate the metabolic, epigenetic, and epidemiological effects of RUTF supplementation on SAM survivors, and to devise treatment options for SAM that are based on nutrient-dense foods that approximate healthier diets. Development efforts are needed to improve dietary intakes in low-income countries to prevent SAM. Finally, there is an urgent need for long-term evaluations of the individual and combined effects of diverse obesity treatment approaches in young children, with particular emphasis on articulating metabolic and multi-omics pathways mediating observed outcomes.

## Conclusion

This review challenges the diametrical framing of pediatric undernutrition and obesity as discrepant conditions, advocating instead for an integrated, systems-level understanding of nutrition-related disease pathways in childhood. Future investigations and intervention efforts must go beyond compartmentalization of these twin maladies, to target their shared biological mechanisms, particularly with the understanding that both conditions could possibly coexist in households and communities.

## Key References

- Alves JG, Alves LV. Early-life nutrition and adult-life outcomes. J Pediatr. 2024;100(suppl 1):S4-9.
  - ○ Early childhood undernutrition and obesity, when followed by abundant, energy-dense diets, trigger metabolic adaptations that substantially elevate obesity risk and comorbidities in adulthood.
- Kirolos A, Harawa PP, Chimowa T, Divala O, Freyne B, Jones AG, Lelijveld N, Lissauer S, Maleta K, Gladstone MJ, Kerac M. Long-term outcomes after severe childhood malnutrition in adolescents in Malawi (LOSCM): a prospective observational cohort study. Lancet Child Adolesc Health. 2024;8(4):280-9.
  - ○ Adolescents exposed to childhood severe acute malnutrition experienced sustained height deficits with no clear signs of medium-term increased cardiometabolic risk.
- Zhang, Y., Zhou, Y., Cheng, Y. et al. Association of birth and childhood weight with risk of chronic diseases and multimorbidity in adulthood. Commun Med. 2023;3: 105.
  - ○ Low or high birth weight, thinness, obesity, or rapid weight gain or loss in childhood are associated with an increased risk of chronic diseases and multimorbidity in adulthood.

## Data Availability

No datasets were generated or analysed during the current study.

## Abbreviations

BMI: Body Mass Index
DNA: Deoxyribonucleic Acid
DUSP1: Dual Specificity Phosphatase 1
ESAM: Edematous Severe Acute Malnutrition
F/B: Firmicutes to Bacteroidetes ratio
FTO: Fat Mass and Obesity-Associated Gene
GH: Growth Hormone
GPCR: G-Protein Coupled Receptors
GPx3: Glutathione Peroxidase 3
GRP78: Glucose-Regulated Protein 78
H3Ac: Histone H3 Acetylation
H3K20me3: Histone H3 Lysine 20 Trimethylation
H3K4me3: Histone H3 Lysine 4 Trimethylation
H3K9me: Histone H3 Lysine 9 Mono- or Di-Methylation
H3K9me3: Histone H3 Lysine 9 Trimethylation
H4Ac: Histone H4 Acetylation
HDAC: Histone Deacetylase
IDH1: Isocitrate Dehydrogenase 1
IGF-1: Insulin-like Growth Factor 1
IL-10: Interleukin 10
IL-18: Interleukin 18
IL-6: Interleukin 6
KAZN: Kazrin gene
KMT1A: Lysine Methyltransferase 1 A (also known as SUV39H1)
LPIN1: Lipin 1
LXRα: Liver X Receptor Alpha
MUAC: Mid-Upper Arm Circumference
MAFLD: Metabolic Dysfunction–Associated Fatty Liver Disease
NESAM: Non-Edematous Severe Acute Malnutrition
NF-κB: Nuclear Factor kappa-light-chain-enhancer of activated B cells
PDE3A: Phosphodiesterase 3 A
PDGFR: Platelet-Derived Growth Factor Receptor
PMN: Peripheral Mononuclear Cells
PPARG: Peroxisome Proliferator-Activated Receptor Gamma
RNA: Ribonucleic Acid
SAM: Severe Acute Malnutrition
SCFA: Short Chain Fatty Acids
SIRT1: Sirtuin 1 Gene
SLC17A9: Solute Carrier Family 17 Member 9
T3: Triiodothyronine
T4: Thyroxine
TDH: L-Threonine Dehydrogenase
TGF-β: Transforming Growth Factor Beta
TNF: Tumor Necrosis Factor
TSH: Thyroid-Stimulating Hormone
WHZ: Weight-for-Height Z-score
mTOR: Mechanistic Target of Rapamycin
